# Nurses' perceptions of quality of work life in private hospitals in Enugu, Nigeria: A qualitative study

**DOI:** 10.3934/publichealth.2022050

**Published:** 2022-11-01

**Authors:** Daniel Ogbuabor, Nwanneka Ghasi, Raymonda Eneh

**Affiliations:** 1 Department of Health Administration and Management, Faculty of Health Sciences and Technology, University of Nigeria Enugu Campus, Enugu, Nigeria; 2 Department of Management, Faculty of Business Administration, University of Nigeria Enugu Campus, Enugu, Nigeria; 3 Department of Health Systems and Policy, Sustainable Impact Resource Agency, Enugu, Nigeria

**Keywords:** quality of work life, nurses, nursing, work-family life balance, work design, work context, work relevance, work world

## Abstract

Despite being essential for retaining nurses, not much is known about nurses' quality of work life (QWL) in private hospitals in sub-Saharan Africa, including Nigeria. We explored nurses' perceptions of QWL, factors influencing it, how it affects motivation, and strategies for its improvement. The study was conducted in seven private hospitals in Enugu, Nigeria. The design was qualitative, using focus group discussion (n = 7) with registered nurses (n = 66) purposively selected using maximum variation sampling and the inclusion criteria. Data were analyzed using verbatim transcription and thematic analysis. The nurses understood QWL from work-family life, work design, work context and work world perspectives. Opportunities for skill acquisition, resource availability, helpfulness from colleagues, and a hygienic work environment improved the QWL and motivation of nurses. Work-family life factors including caring obligations, night shifts, long hours, burnout, and inappropriate leave policies; work design factors including declining autonomy, inadequate staffing, and a high workload; work context factors consisting of a lack of participatory decision-making, blaming nurses for gaps, restrictive training policy, limited training opportunity, and insecurity; and work world factors related to poor remuneration, poor community view of nursing and ease of job termination undermined QWL and demotivated nurses. Strategies identified by the nurses to improve QWL included improving staffing, vacation, care coordination, supportive supervision, teamwork, promotion, participatory decision-making, training opportunities, timely hand-over of shifts, job recognition, and compensation. The quality of nursing work life in private hospitals in Enugu needs improvement. Quality improvement programs addressing the barriers to nurses' QWL are warranted.

## 1. Introduction

Increasing attention has been paid to the quality of work life (QWL) in healthcare settings as a mechanism for resolving persisting health workforce challenges [1]–[4]. Nurses' QWL is defined as “the degree to which registered nurses are able to satisfy important personal needs through their experiences in their work organization while achieving the organization's goals” [5]. QWL posits that people are the most critical resource in an organization and should be treated with dignity and respect [6],[7]. QWL focuses on workplace improvement based on a dual agenda of employee well-being and organizational performance [6]. An improved QWL is essential to recruit and retain nurses, increase their productivity and organizational commitment, enhance effectiveness of the patient care system and ensure greater patient satisfaction [8]–[11].

QWL comprises four dimensions: work-family life balance (WFB), work design, work context and work world [5]. Studies examining WFB investigated how the interaction between work and personal life affects the well-being of nurses. The evidence indicates that the lack of a support structure for WFB practices negatively impact family and friend relationships [8],[12]–[14]. Nurses lack energy after work due to exhaustion from high workloads [13]. Caregiving obligations at home are limited by staff shortages, inflexible shift schedules and long work hours [8],[13],[14]. Loss of vacation time results from inadequate staffing and demands of rotational shifts [8]. Female nurses had a sense of inadequacy and experienced guilt toward their colleagues for not being able to go to work for family-related reasons and needing to ask for help from colleagues [15],[16]. Female nurses juggle managing their children, house and self-needs [15],[16]. With childcare obligations, nurses face more responsibilities at home and are less willing to report to work during a pandemic [17].

Work design encompasses the actual work nurses do and how the immediate work environment, such as autonomy at work, the workload and human resource availability; the time to do a job, non-nursing tasks and receiving assistance at work affect the well-being of nurses and organizational performance [5]. Evidence indicates that an excessive workload [3],[8],[18]–[20], lack of staff [19],[21]–[23], long work hours [24],[25], weekend schedules [24], emotional exhaustion [18],[26], conflicting priorities and diffuse responsibilities [6] and a lack of autonomy [23] negatively impact the quality of nursing work life. Nurses assumed various non-nursing roles at different levels, backstopping other health professionals to offer the best to their patients and their organization [8],[27]. Assisting a colleague and the willingness to accomplish tasks were two drivers of overtime and long hours in Cambodia [25].

Work context embodies the practice settings in which nurses work and the impact of the work environment on both nurse and patient systems, including management patterns, style of supervision, resource availability, co-worker relationships, promotional opportunities, feedback and a conducive working environment [5]. Helpfulness and assistance from colleagues were associated with a high quality of nursing work life [13],[28],[29]. Nonetheless, blame culture [30], a lack of sufficient resources [6],[11],[18],[21],[22],[29],[31], a lack of supportive organizational leadership and policies [3],[20],[21],[28],[32], a lack of support from the nursing supervisor [3],[21],[31], medical dominance [6],[31], a lack of participatory decision-making [6],[20],[32], a lack of promotion [3],[32], few professional training opportunities [32], a lack of personal protective equipment [19],[20],[29] and an unsafe environment [28],[31] contributed to a low QWL among nurses.

The work world entails public perception of the work nurses do and the effects of societal influences on the practice of nursing, including the image of nursing, job security, adequate compensation and benefits, the labor market and belief in nursing [5]. Qualitative studies investigating the social relevance of work among nurses are scarce. Two studies found that job pride improved the quality of nursing work life [22],[28]. In the private sector, inadequate remuneration [29],[32] and reduced opportunities for continuing professional development constrained the quality of nursing work life [32]. In the public sector, low compensation [8], a lack of financial incentives [3], low public perception [8],[33],[34] and a loss of job pride [8] are associated with a low quality of nursing work life.

Despite the private health sector delivering more than 60% of health care in Enugu State, Nigeria [35], little attention has been paid to QWL and its associated factors among their nurses. The private hospitals face considerable difficulties in attracting and retaining health care providers, especially registered nurses, because of better conditions of service in the public sector. As a result, the bulk of nursing staff in private hospitals are in-service trained nurse assistants. However, large private hospitals seem able to attract and retain more registered nurses in Enugu metropolis. There is a need to identify the human resource management practices and work climate that enable such private hospitals to retain registered nurses even when they could perhaps earn more elsewhere.

Therefore, this study assessed registered nurses' understanding of the concept of QWL, perceptions of their QWL, factors affecting the quality of nursing work life, how quality of nursing work life influences their motivation and strategies for improving nurses' QWL in private hospitals in Enugu metropolis, Nigeria. This information would be useful to decision-makers and providers in developing, implementing and monitoring human resource management practices to improve nurses' well-being and organizational performance in private hospitals in low resource settings.

## 2. Materials and methods

### 2.1. Study area

The study was conducted in Enugu metropolis, Enugu State, Nigeria. Enugu State has a three-tier health system comprising primary, secondary and tertiary subsystems. Private and public health sectors co-exist. Private hospitals are registered and regulated by the Enugu State Ministry of Health. The metropolis was chosen because of its high concentration of large privately funded hospitals. Seven big private hospitals were purposively chosen for the study. The big private hospitals were selected because they provide general and specialist medical, surgical, gynecological/obstetric and pediatric services and employed registered nurses.

### 2.2. Study design

The study adopted a qualitative, exploratory research design to collect detailed information from focus groups of registered nurses. This design was chosen because nursing QWL might be best understood through the meaning that participants assign to their experiences since not much is known about nursing QWL in Nigerian private hospitals.

### 2.3. Study participants

The target population was all registered nurses currently employed in the selected private hospitals in Enugu metropolis, Nigeria. Registered nurses were included in the study if they were employed on a full-time basis and had worked for a minimum of one year in the hospital, as they were then more likely to have experienced various aspects of work life, which constituted the central foci of this study. The study excluded nurse assistants, nurses who had not worked up to a year in the hospital, nurses on leave (sick, maternity, annual and casual) and nurses employed on a part-time basis.

Purposive, non-probability sampling was used to select participants from the nurses employed in the hospitals who met the inclusion criteria for the study. The head of nursing services facilitated participant recruitment in each hospital. Eligible registered nurses were identified, informed about the study and invited to participate. Participation was based on the nurses' consent. Once recruited, registered nurses were given a time and place for the focus group discussion (FGD). We recruited 8 to 10 registered nurses per hospital to foster inclusion of all group members and yield a diversity of the information provided.

### 2.4. Data collection

Seven FGDs were held with 66 participants in seven hospitals using a discussion guide. FGD was deemed appropriate because it has been used to evaluate participants' experiences within healthcare, it enables participants to concentrate on research objectives and react to what others said and it increases the range of views on phenomena of interest [36]. The discussion guide was based on the conceptual framework and research objectives of this study. The guide consisted of questions on nurses' understanding of the concept of QWL, perceptions of their work-family life balance, work design, work context and the social relevance of their work. Other topics included in the guide were factors affecting nursing QWL, the influence of QWL on the motivation of nurses and strategies for improving nursing QWL. The FGDs were held in a quiet room within each hospital. Each FGD lasted about 60–90 minutes, was conducted in English, and was audio recorded.

### 2.5. Data analysis

All interview tapes were transcribed verbatim. A thematic framework approach was adopted, in which data were classified and organized based on themes, concepts and emergent patterns. The thematic analysis involved five steps: developing codes, coding data, sorting the data by matching the codes with segments of text, connecting the codes and identifying patterns and summarizing and synthesizing the data. Codes were developed by following a process of deduction based on the conceptual framework of the study. The codes were defined in a codebook to create a common understanding of each code. NVivo 11 software was used by two independent researchers to code meaningful fragments in the text to the themes. Data were sorted and grouped together, and patterns were identified based on dimensions of QWL. We summarized and synthesized the data while retaining significant statements and phrases by participants to enable interpretation of meaning.

### 2.6. Ethical considerations

Ethical approval to conduct the study (MH/MSD/REC20/140) was obtained from the University of Nigeria Teaching Hospital Research Ethics Committee, Enugu, Nigeria. Written, informed consent was obtained from all participants for participation and audio recording of the FGDs.

## 3. Results

### 3.1. Basic characteristics of participants

The socio-demographic characteristics of participants are shown in Table 1. All participants were female nurses drawn from four clinical departments of the hospitals. Most nurses were married. Fifty-two percent of nurses have worked for less than five years in the hospital. More than half of the nurses had dependent children or relations.

**Table 1.** Basic characteristics of focus group participants.

Socio-demographic factors		N = 66	Percentage (%)
Age	21–29	16	24
	30–39	27	41
	40–49	13	20
	50–59	10	15
Marital status	Never married	22	33
	Married	44	67
Number of dependents	None	22	33
	≤2	17	26
	>2	27	41
Tenure	≤5 years	34	52
	>5years	32	48
Current department	Medical	17	26
	Surgery	11	17
	Maternity/Antenatal	18	27
	Newborn	7	11
	GOPD/Accident & Emergency	13	20

### 3.2. Meaning of quality of work life

Participants understood QWL from four main perspectives: work-family life, work design, work context and work relevance perspectives (Table 2).

#### 3.2.1. Work-family life perspective

Nurses understood QWL as balancing the demands from society and their work, including the relationship between their job and their family, social life and caregiving roles, time spent at work and policy on vacation time. “*QWL means how my work affects my family, my vacation, the duration of time I spend in the hospital and at home*” (N3, FGD1 FBH).

**Table 2.** Meaning of QWL among nurses in private hospitals in Enugu, Nigeria, 2021.

QWL dimension	Category	Subcategory
WFB	Policy on vacation time	Impact of work on vacation time
	Working hours	Duration of time at work
	Family needs	Managing personal life and family affairs
	Family needs	Nurses' well-being
	Working hours	Managing social life
Work design	Patient care system	Patient well being
	Patient care system	Responsiveness
	Workload	Excessive work
Work context	Management/leadership	Participation in decision-making
	Management/leadership	Organizational justice
	Management/leadership	Achieving organizational goals
	Management/leadership	Recognition
	Coworker relationships	Interaction with coworkers
Work relevance	Image of nursing	Community view of nursing/Job pride
	Image of nursing	Family view of nursing job
	Adequate and fair pay	Satisfaction with pay

#### 3.2.2. Work design perspective

Nurses also understood QWL from workload and responsive nursing care perspectives. They interpreted QWL as how the “*practice setting affected standard of nursing care delivered to patients*” (N5, FGD2, FBH), and as “*being given the freedom to carry out your jurisdiction without anybody interfering in it*” (N1, FGD4, FBH).

#### 3.2.3. Work context perspective

Nurses' understanding of QWL centered on management and leadership practices, social interaction at work and the conduciveness of their work environment. *“Am I appreciated by the management of the hospital?”* (N6, FGD2, FBH). Most nurses emphasized participation in decision-making and organizational justice: “*QWL means whether nurses are free to say their minds. It involves the challenges we have at workplace and how those challenges are being addressed, whether it is in favour of the nurses or the employer”* (N9, FGD7, PFPH).

#### 3.2.4. Work world perspective

QWL was perceived as appropriate family and community views of nursing as a socially relevant profession and rewarding in terms of remuneration: *“After everything, at the end of the month, am I satisfied with the package they give me?”* (N4, FGD3, PFPH).

### 3.3. Factors affecting quality of work life

#### 3.3.1. Work-family life balance

Four themes emerged from WFB, namely, family and social needs, energy after work, working hours/shifts and vacation policy (Table 3).

Nurses' caregiving responsibilities, social life and family needs often conflicted with their work. Nurses who must attend to family obligations have to ask their colleagues to take their shifts. Night shifts deprived nurses of time to care for their families. Instead of triple shifts, most hospitals run double shifts. Nurses spend long hours working in the hospital, get home burnt out and unable to attend to family needs. “*Doing the work, at the end of the day, reaching your house, even to sleep or to eat becomes a problem”* (N3, FGD3, PFPH). Moreover, with the COVID-19 pandemic, many private hospitals increased their working days. Yet, private hospitals, which do not observe all public holidays, have reduced the maternity leave period and have a rigid casual leave policy.

*“During COVID-19, they wanted us to work for six days in a week and have only a day off. After the protests, the hospital reverted to two days off-duty in a week”* (N6, FGD4, FBH).

#### 3.3.2. Work design

Declining autonomy, a heavy workload, interruption of nursing care and the performance of non-nursing tasks emerged as themes under work design (Table 3).

Exposure to a variety of challenging cases improved nursing QWL. However, limited use of initiative reduced nurses' autonomy and control at work. A heavy workload, resulting from inadequate staffing, contributed to poor nursing QWL. Nurses were discontented with interruptions of nursing care by other professionals and owner-managers of private hospitals. *“Some private hospitals will turn their registered nurses to cleaners, they will ask you to clean the floor, or clean the MD's office”* (N4, FGD7, PFPH).

Night shifts and excessive workloads are associated not only with emotional exhaustion, especially among nurses with caregiving responsibilities at home, but also with the inability of nurses to meet patients' nursing care demands. Nurses noted how frustrating it was to have a decreased feeling of competence and achievement at work resulting from poor nursing care planning or a lack of teamwork. *“We had a lot of patients, that it made almost everyone to fall ill, each and everyone, even the doctors, the pressure was much, you will be running around”* (N9, FGD7, PFPH).

#### 3.3.3. Work context

Leadership/management practices, coworker relationships, growth opportunities and a safe and healthy work environment were the themes related to work context.

Nurses indicated that, apart from the unavailability of personal protective equipment in the early phase of COVID-19, the availability of other working materials and planned preventive maintenance of equipment improved nurses' QWL. *“Unlike doctors, they started giving us masks after we protested, though this was not 95%”* (N1, FGD6, FBH). Nurses do not participate in decision-making due to rigid a organogram. The head of nursing services lacks a voice in the hospital management. Even when hospitals have opinion boxes, suggestions from nurses are not usually considered in decisions. Still, nurses are blamed for most gaps in patient care. *“Everything that goes wrong here is blamed on the nurse”* (N5, FGD3, PFPH). Promotions are not based on performance appraisal. While one group of nurses believed supervision is supportive, the other claimed nurses are weakly supervised. Querying nurses when patients purchase drugs outside of the hospital pharmacy, even in the face of stockouts, hampers nursing QWL.

**Table 3.** Factors influencing nursing QWL in private hospitals in Enugu Metropolis, Enugu State, Nigeria, 2021.

Domain	Theme	Sub-theme
WFB	Family needs	Caregiving responsibilities / family obligations
	Work hours	Night shifts
		Working longer hours
	Energy after work	(Burnout) Exhaustion due to long hours and excessive work
	Policy on vacation time	Non-observance of public holidays
		Reduced maternity leave period
		Rigid casual leave policy implementation
Work design	Autonomy and control at work	Declining autonomy
	Interruptions	Interference with nursing care
	Non-nursing tasks	Doing the job of porters/cleaners and doctors
	Patient care system	Skill acquisition
	Inadequate staffing / patient care system	(Burnout) Depersonalization
	Type of shift / workload	(Burnout) Emotional exhaustion
	Time to do work	(Burnout) Decreased personal achievement
Work context	Management	Availability of resources – equipment, drugs and supplies
		Planned preventive maintenance of equipment
		Use of hospital organogram
		Organizational justice / blaming nurses for gaps
		Lack of performance evaluation/appraisal
		Weak supportive supervision
		Drug-use policy
	Coworker relationships	Interaction with nursing colleagues
		Assistance from nursing colleagues
		Poor socialization
		Interaction with nurse managers/management
	Growth opportunities	Restrictive training policy
		Limited opportunity for continuing professional development
	Work environment	Hygienic environment
		Regular public utilities in the hospital
		Insecurity around hospital location
Work world	Adequate and fair compensation	Poor remuneration
	Image of nursing	Poor community view of nursing
	Belief in nursing	Pride as nurses
	Job security	Ease of job termination
	Labor market	Turnover intention

Cordial relationships with and receiving assistance from nursing colleagues improved nursing QWL. *“There is no intimidation by virtue of rank or whatever”* (N3 FGD3 PFPH). Nurses are also happy with the hygienic environment and regular supply of utilities. Nonetheless, nurses indicated that the training policy that restricts nurses from enrolling for in-service academic programs constrained their QWL. Job insecurity among nurses undergoing self-sponsored continuing professional education negatively affected their QWL. *“About ten of them or more were sacked from work instantly”* (N5 FGD2 FBH). Some nurses noted that poor security around the hospital location led to poor QWL, as *“nurses are often attacked by arm robbers along their way home at night”* (N3 FGD1 FBH). Delays in hand-overs between nurses on afternoon and night shifts exposed nurses to such criminal attacks.

#### 3.3.4. Work world

Poor remuneration, poor community view and ease of job termination were identified as constraints to nursing QWL. Nurses were discontented with a lack of increment in salary over many years. *“I have worked here for several years, but the salary remains the same”* (N3 FGD5 FBH). Worse still, at the peak of COVID-19, nurses received half salaries while working full-time. Nurses also lamented about punitive salary cuts, while there are no incentives for overtime.

Even though professional pride positively influenced nursing QWL, most nurses stated that poor community views of nurses compared to doctors and societal degrading orientation about the nursing profession negatively affected their QWL. *“When we [nurses] advise them, they do not take it. They want the doctor to say it before they carry out our instructions”* (N1 FGD4 FBH). Bill handling also contributed to a poor image of nurses.

Ease of job termination was identified by most participants as negatively affecting nursing QWL. As a result, almost all nurses are looking for public sector jobs. A nurse queried: *“what will it benefit me if I put up 10 years, at the end of the 10 years, I will go home with nothing to fall back on?”* (N3 FGD7 FPH).

### 3.4. Influence of quality of nursing work life on motivation

Most QWL factors identified by nurses do not motivate them. In contrast, opportunities for skill acquisition, the availability of resources, planned preventive maintenance, interaction with colleagues, assistance from colleagues, a hygienic work environment, regular public utilities and job pride motivated them (Table 4).

### 3.5. Strategies for improving quality of nursing work life

Nurses identified improved staffing, revision of personnel policy, improved autonomy, care coordination, participatory decision-making, performance appraisal, a flexible training policy, regular promotion, improved salaries and societal recognition as effective strategies to improve nursing QWL (Table 5).

**Table 4.** Influence of QWL on the motivation of nurses in private hospitals in Enugu, 2021.

QWL dimension	Sub-dimensions	Themes	Effect on motivation
WFB	Family needs	Caregiving obligations	Demotivates
	Work hours	Night shifts	Demotivates
		Working longer hours	Demotivates
	Energy after work	Exhaustion due to long hours and excessive work	Demotivates
	Policy on vacation time	Non-observance of public holidays	Demotivates
		Reduced maternity leave period	Demotivates
		Rigid casual leave policy implementation	Demotivates
Work design	Autonomy and control at work	Declining autonomy	Demotivates
	Interruptions	Interference with nursing care	Demotivates
	Non-nursing tasks	Doing the job of porters	Demotivates
	Patient care system	Opportunity for skill acquisition	Motivates
	Staffing	Inadequate staffing	Demotivates
	Workload	Work demand	Demotivates
Work context	Leadership and management	Availability of resources – equipment, drugs	Motivates
		Planned preventive maintenance of equipment	Motivates
		Use of hospital organogram	Demotivates
		Disturbance handling / blaming nurses for gaps	Demotivates
		Lack of performance evaluation/appraisal	Demotivates
		Weak supportive supervision	Demotivates
		Drug-use policy	Demotivates
	Coworkers	Interaction with nursing colleagues	Motivates
		Assistance from nursing colleagues	Motivates
		Poor socialization	Demotivates
		Interaction with nurse managers/management	Demotivates
	Growth opportunity	Restrictive training policy	Demotivates
		Limited opportunity for continuing professional development	Demotivates
	Safe and health work environment	Hygienic environment	Motivates
		Regular public utilities in the hospital	Motivates
		Insecurity around hospital location	Demotivates
Work world	Adequate and fair compensation	Poor remuneration	Demotivates
	Image of nursing	Poor community view of nursing	Demotivates
	Belief in nursing	Pride as nurses	Motivates
	Job security	Ease of job termination	Demotivates
	Labor market	Turnover intention	Demotivates

**Table 5.** Strategies for improving QWL on the motivation of nurses in private hospitals in Enugu, Nigeria, 2021.

QWL dimension	Sub-dimensions	Themes	Strategies proposed
WFB	Family needs	Caregiving obligations	Improve staffing
	Work hours	Night shifts	
		Working longer hours	
	Energy after work	Exhaustion due to long hours and excessive work	
	Policy on vacation time	Non-observance of public holidays	Revise personnel policy
		Reduced maternity leave period	
		Rigid casual leave policy implementation	
Work design	Autonomy and control at work	Declining autonomy	Allow nurses to use initiative under supervision
	Interruptions	Interference with nursing care	Coordination with other clinical departments
	Non-nursing tasks	Doing the job of porters	Improve staffing
	Staffing	Inadequate staffing	Improve staffing
	Workload	Work demand	Improve staffing
Work context	Management	Disturbance handling / blaming nurses for gaps	Coordination with other clinical departments; Stop blaming nurses
		Lack of performance evaluation/appraisal	Introduce annual appraisal; appreciation by management
		Weak supportive supervision	Improve staffing
		Drug-use policy	Allow use of essential drugs bought outside of the hospital
	Coworkers	Poor socialization	Create opportunities for social interaction
		Interaction with nurse managers/management	Cordial relationships
	Growth opportunity	Restrictive training policy	Flexible training policy
		Limited opportunity for continuing professional development	Promotion, sponsorship to conferences and the bonding of staff on hospital-supported, in-service training
	Safe and health work environment	Insecurity around hospital location	Timely hand-over of shifts
Work world	Adequate and fair compensation	Poor remuneration	Improve salaries
	Image of nursing	Poor community view of nurses	Societal recognition of nurses
	Job security	Ease of job termination	Adhere to personnel policy

## 4. Discussion

This study has provided insight into the QWL of nurses in private hospitals in Enugu, Nigeria, their concepts and perceptions, factors influencing their perceptions, how QWL affects motivation and strategies for its improvement.

Registered nurses in private hospitals have a good understanding of the concepts of QWL in this study. They perceived QWL from the four dimensions of Brooks' quality of nursing work life framework: work-life/home-life balance, work design, work context and work world [5]. From the work-home life balance perspective, *“impact of work on vacation”, “time spent at work”, “managing personal and family life”* and *“nurses' well-being”* were key concepts highlighted. Pertaining to work design, the key concepts described include *“patient well-being”*, *“responsiveness to patient care”* and *“excessive work”*. Major concepts identified within work context are *“participation in decision-making”*, *“organizational justice”*, *“organizational goals”* and *“interaction with co-workers”*. Participants identified *“job pride”*, *“community view of nursing”* and *“satisfaction with pay”* as main concepts pertaining to the work world. Vagharseyyedin and colleagues argue that concept analysis is useful in clarifying the meaning of QWL based on employees' experiences at work [37]. Nurses' perception of their work climate is important in their understanding of QWL.

The study revealed that work-home life balance factors negatively affect the quality of nursing work life and motivation. Caring obligations were critically important to the nurses but were hampered by night shifts, long working hours and reduced vacation opportunities. The findings are in line with previous studies that found that caregiving responsibilities, inflexible shift schedules, long work hours, rotational shifts and loss of vacation time contributed to low QWL among nurses [8],[13],[14]. As reported in a preceding work, these factors alter the balance between work and family life, trigger a sense of inadequacy and guilt toward their dependents and hospitals and compel nurses to juggle managing their children, house and self-needs [15],[16]. To improve work-home life balance, our findings reinforce a need for staffing to match the workload and rotational shifts, as well as revision of the vacation policies to ensure the observance of public holidays and a longer maternity leave period. Improved staffing might mitigate the negative effects of WFB by reducing the number of night shifts, increasing the rotational shifts and enhancing opportunities for vacation.

This study's findings indicate that opportunities for skill acquisition and use positively affected quality of nursing work life, which is consistent with nurses' experiences of more challenging tasks and opportunities for diverse clinical roles in Ugandan private health facilities [38]. Nonetheless, the declining autonomy [23], interference with nursing tasks [6], non-nursing tasks [8],[27], inadequate staffing [19],[21]–[23] and high work demands [3],[8],[18]–[20] found in previous studies were also found to negatively affect nursing QWL in the current study. Burnout emerged as an important quality of nursing work life concern in this study. Nurses revealed their experiences of three types of burnout: emotional exhaustion, depersonalization and decreased personal achievement at work. Night shifts and an excessive workload are associated with emotional exhaustion, especially among nurses with family needs. Depersonalization is related to inadequate staffing and the inability of nurses to meet patients' nursing care demands. Nurses noted how frustrating it was to have a decreased feeling of achievement at work resulting from poor nursing care planning or a lack of teamwork. Improved staffing, allowing nurses to use initiative under supportive supervision, improving coordination with other departments and fostering teamwork are meaningful strategies that might improve the work design. Adequate nurse staffing, focus on nursing tasks and non-interference with nursing care will improve the nurse-to-patient ratio, decrease workloads and limit nurses' experiences of burnout [39].

Even though a conducive working environment improves quality of nursing work life, the findings of this study highlighted several gaps in the work context which undermine the QWL and motivation of nurses. Nurses were blamed for most gaps in patient care, which is consistent with the blame culture found in prior studies [30],[40]. Also, nurses lacked voice in the decision-making in private hospitals, as was found in other studies [6],[20],[32]. Consistent with existing evidence [3],[21],[31], the support from nursing supervisors was mostly lacking. Yet, promotion opportunities were inadequate and not based on performance evaluation, which is similar to existing scholarship [3],[32]. Equally, nurses attending continuing professional development programs risk losing their jobs due to restrictive training policies [32]. In the same vein, insecurity led to a poor quality of nursing work life, resulting in the unsafe environment found in preceding studies [28],[31]. As was found elsewhere [19],[20],[29], nurses lacked personal protective equipment during the COVID-19 pandemic, which undermined their QWL. Nurses emphasized opportunity for professional growth, a conducive work environment, good nurse-supervisor interaction and participation in decision-making as important to improvement in their QWL.

Despite the finding that professional pride positively influenced quality of nursing work life, the findings indicated that poor remuneration, poor community view of nurses, ease of job termination and turnover intention worsened quality of nursing work life. While professional pride motivated nurses, poor remuneration, poor public perception and ease of job termination demotivated them. Evidence from previous studies supports our findings that nurses are discontented with their pay [3],[8],[29],[32], low public perception [8],[33],[34] and ease of job termination [37]. Work world strategies identified by nurses to improve their QWL are improved salaries, adherence to personnel policy to guarantee job security and societal recognition of nursing contributions to the patient care system. The need to properly value nurses and pay them well were expressed by participants. Improving pay and benefits for nurses have been suggested by nurses in other studies as a way of improving their quality of nursing work life [3],[8].

This study provides rich insight into the context of human capital management in privately funded hospitals. To our knowledge, this is the first study of the quality of nursing work life in private hospitals in Nigeria. However, this study represented the views of participants from a small number of private hospitals in one Nigerian state. Therefore, the findings cannot be generalized to other private health institutions in other states or publicly owned hospitals in Nigeria. As the study was done using purposive sampling, it is possible that there may be other nurses who are knowledgeable enough but were not part of the study. Similarly, the perceptions of study participants may have been shaped by social desirability bias; in which case, responses given might not correspond to actual experiences of nursing QWL. Interestingly, data from the FGDs were consistent and saturation seemed to have been achieved.

## 5. Conclusions

The purpose of this study, which was to understand the perceptions and experiences of nurses concerning their QWL in private hospitals, has been achieved. Specifically, the study uncovered the meaning nurses gave to their experiences of QWL, identified context-specific factors influencing quality of nursing work life, assessed how various dimensions of QWL affected the motivation of nurses and yielded strategies for improving their QWL. Implications of these findings for healthcare management research and nursing practice have been indicated. It is concluded that this work builds on existing literature on QWL and, for the first time, provides insight at a very detailed level, into what constitutes quality of nursing work life in private hospitals in Nigeria.
